# Elastography Versus B-Mode Lung Ultrasound for the Diagnosis of Iatrogenic Pneumothorax: An Observational, Monocentric, Prospective Study

**DOI:** 10.3390/jcm14092978

**Published:** 2025-04-25

**Authors:** Gian Piero Bandelli, Ilaria Bassi, Alessandro Zanforlin, Riccardo Inchingolo, Martina Ferioli, Alessandro Cipolli, Arianna Johanna De Grauw, Marco Ferrari, Thomas Galasso, Filippo Natali, Tommaso Abbate, Francesco Buia, Domenico Attinà, Fabio Niro, Luciana Ingraldi, Elena Nardi, Luigi Lovato, Piero Candoli

**Affiliations:** 1 Interventional Pulmonology Unit, IRCCS Azienda Ospedaliero-Universitaria di Bologna, 40138 Bologna, Italy; gianpiero.bandelli@aosp.bo.it (G.P.B.);; 2 Alma Mater Studiorum, Department of Medical and Surgical Sciences (DIMEC), University of Bologna, 40138 Bologna, Italy; 3 Respiratory and Critical Care Unit, IRCCS Azienda Ospedaliero-Universitaria di Bologna, 40138 Bologna, Italy; 4 Service of Pulmonology, Health District of Bolzano (SABES-ASDAA), Teaching Hospital of Paracelsus Medizinischen Privatuniversität, 39100 Bolzano-Bozen, Italy; 5 UOC Pneumologia, Fondazione Policlinico Universitario A. Gemelli IRCCS, 00168 Roma, Italy; 6 Division of Thoracic Surgery, IRCCS Azienda Ospedaliero-Universitaria di Bologna, 40138 Bologna, Italy; 7 Pulmonology Unit, Morgagni Pierantoni Hospital, 47121 Forlì, Italy; 8 Pediatric and Adult CardioThoracic and Vascular, Oncohematologic and Emergency Radiology Unit, UOC di Radiologia, Radiologia Cardiovascolare, IRCCS Azienda Ospedaliero-Universitaria di Bologna, 40138 Bologna, Italy; 9 Epidemiology and Statistics Unit, IRCCS Azienda Ospedaliero-Universitaria di Bologna, 40138 Bologna, Italy

**Keywords:** chest ultrasound, elastography, pneumothorax, iatrogenic pneumothorax, B-mode

## Abstract

**Background**: Thoracic ultrasound (TUS) has emerged as a viable alternative of computed tomography (CT) for pneumothorax diagnosis. Ultrasound elastography (USE), a technique assessing tissue elasticity, has recently been proposed as a novel tool for pneumothorax evaluation. **Methods**: This prospective, monocentric, observational study aimed to compare the diagnostic accuracy of static and dynamic USE with TUS in detecting iatrogenic pneumothorax after CT-guided transthoracic needle aspiration (TTNA). **Results**: Thirty-two patients were enrolled, with pneumothorax confirmed via CT in 40.63% of cases. The results showed that elastographic-mode images had significantly higher sensitivity (76.9% vs. 21.2%, *p* < 0.001) and improved positive and negative predictive values (67.8% vs. 52.4%, *p*-value 0.01, 82.6% vs. 61.7%, *p*-value < 0.001, respectively), compared to B-mode images. Concordance between expert and non-expert evaluators was also higher for elastographic images, suggesting improved interpretability. However, dynamic USE did not demonstrate a statistically significant advantage over B-mode videos. **Conclusions**: These findings suggest that USE may enhance static ultrasound-based pneumothorax detection and provide an objective imaging marker for reports. Further multicenter studies are needed to confirm these findings and explore the potential role of USE in other settings.

## 1. Introduction

Pneumothorax is a clinical condition that requires rapid and effective diagnosis and clinical assessment. It is classified as primary and secondary. Primary pneumothorax occurs without an apparent cause and in the absence of significant lung disease. Secondary pneumothorax occurs in the presence of existing lung disease and may be caused by physical chest trauma or as a complication of a medical or surgical procedure [1]. Symptoms typically include chest pain and dyspnea. Diagnosis of pneumothorax requires a chest X-ray (X-ray) or computed tomography (CT) scan. In particular, the chest CT scan is the gold standard for recognizing pneumothorax [2,3].

Thoracic ultrasound (TUS) has emerged as a viable alternative for the diagnosis of pneumothorax with high sensitivity, particularly in cases of trauma, iatrogenic pneumothorax (e.g., after image-guided biopsy), or critical care settings [4]. The specific TUS features are the following: a lack of lung sliding, the absence of vertical artifacts, and the identification of a “lung point” [5]. Lung sliding is the movement of the pleural line during respiration, and its identification rules out pneumothorax in that area. Lung sliding can be assessed both in B-mode and in M-mode. However, the absence of lung sliding is not specific for pneumothorax. Vertical artifacts (often reported as “B-lines”) are projected distally from the pleural line due to imperfections on the lung surface. The presence of vertical artifacts rules out pneumothorax, but their absence does not confirm pneumothorax diagnosis. The “lung point” is an ultrasound sign that locates the junction of the pneumothorax and the normal lung sliding. Among the dynamic signs identified via TUS, the “lung point” is the most specific indicator of pneumothorax, with specificity approaching 100% and a sensitivity of up to 75%, even in cases of radiographically occult pneumothorax [6]. However, a lung point is only visible in partial pneumothoraxes and is dependent upon the patient’s position [5].

Ultrasound elastography (USE), a technique originally developed to assess tissue elasticity and stiffness alterations in pathological conditions, offers a novel approach to pneumothorax evaluation. This technique, developed in the 1990s, detects tissue deformation either following light compressions from an ultrasound probe or using shear wave stimuli generated by ultrasound [7,8]. Elastography is commonly used for the evaluation of the liver, particularly in the assessment of liver fibrosis and cirrhosis. By quantifying tissue stiffness, elastography provides insights into the degree of fibrosis, helping clinicians assess the severity of liver disease and monitor disease progression over time [9]. Besides liver assessment, elastography also has applications in other organs, such as the pancreas and kidneys, where stiffness measurements can indicate the presence of diseases such as cancer or fibrosis, respectively [10].

In respiratory medicine, elastography has been reported to be evaluated for the study of several conditions, such as interstitial lung diseases, chronic obstructive pulmonary disease, and pulmonary edema. Additionally, elastography can be used during endobronchial ultrasound (EBUS). Elastography, combined with EBUS, can assess tissue stiffness during the procedure, providing additional diagnostic information. This integration enhances the ability to differentiate between benign and malignant lesions, as cancerous tissues are often stiffer than surrounding healthy tissue [11].

**Figure 1:**
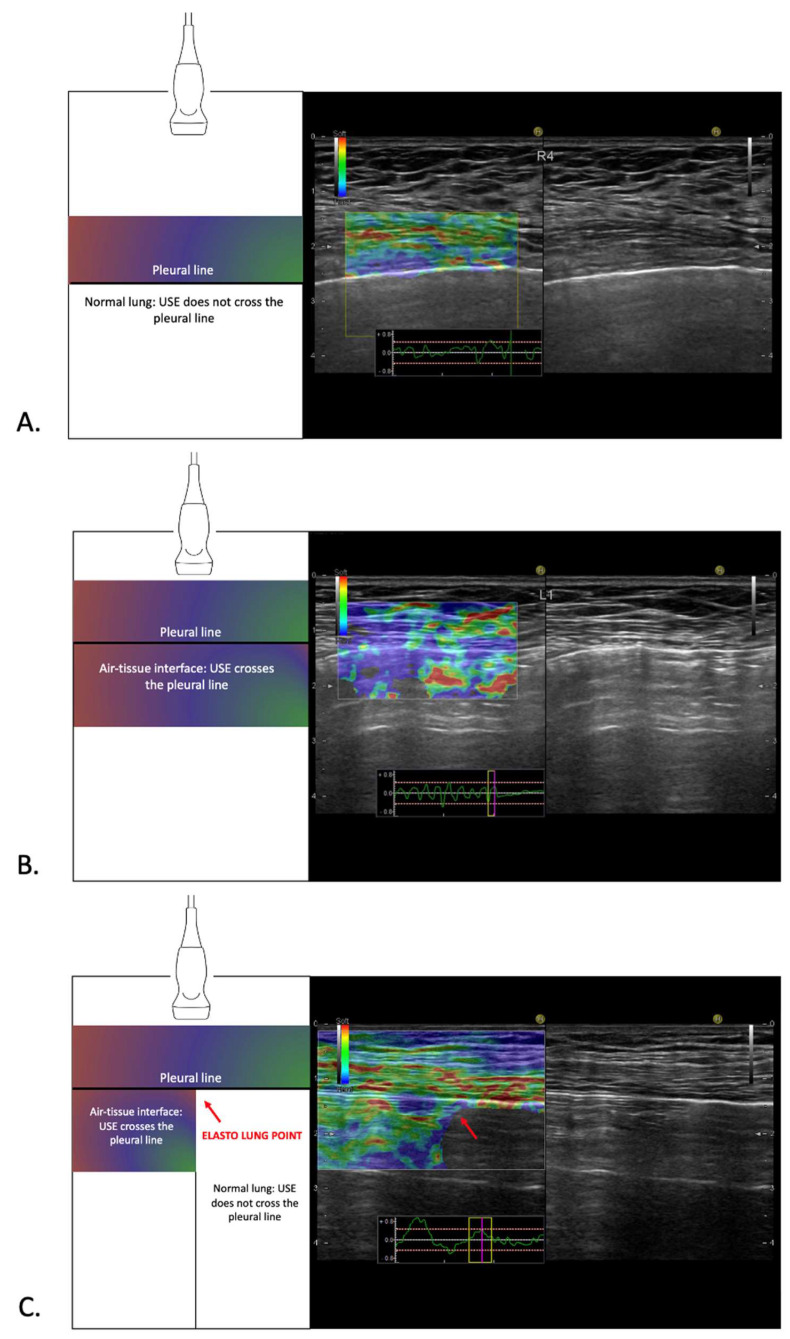
Examples of US elastography. On the left, a schematic representation of the elastographic pattern is shown. On the right, a dual-view screen displays the B-mode ultrasound on the right and the elastography view in the center. (**A**) Normal lung: the color stops at the pleural line. (**B**) Pneumothorax: the color crosses the pleural line. (**C**) Elasto-lung point: in the elastography scan, ultrasound elastography (USE) creates a distinct boundary separating the normal lung parenchyma (on the right of the red arrow, where the color stops at the pleural line) from the air column of the pneumothorax (on the left of the red arrow, where the color crosses the pleural line).

A 2020 observational study described the possibility to apply USE to pneumothorax detection, demonstrating its ability to identify the “lung point” through distinctive stiffness patterns. Specifically, applying USE to a normal lung produces a superimposed elastographic scan in which the elastographic color does not cross the pleural line (Figure 1A). In contrast, applying USE in the pneumothorax region creates a fully colored scan, as echoes penetrate the air–tissue interface (Figure 1B).

Consequently, in the pneumothorax region, the elastographic color crosses the pleural line. This results in a sharp, clear line dividing the normal lung parenchyma from the air column of the pneumothorax, called the “elasto-lung point” (Figure 1C) [12]. This sign can be seen both in static (image) and in dynamic (video) acquisitions. In Figure 1, both the elastographic example and the corresponding US image are displayed together, providing a more comprehensive view of the different scans.

To our knowledge, there are no studies that have investigated the diagnostic accuracy of chest elastography compared to other methods, such as conventional lung ultrasound and chest CT, in the identification of pneumothorax.

Basing on these findings, in order to define the possible added value of elastography in pneumothorax detection, we designed a multicentric prospective study to evaluate the sensitivity, specificity, and diagnostic accuracy of both static and dynamic USE compared to TUS in detecting iatrogenic pneumothoraxes after (CT)-guided transthoracic needle aspiration (TTNA). We also tried to assess the concordance index between expert and non-expert operators in the use of lung ultrasound in B-mode and USE for the recognition of pneumothorax, normal lung, lung point, and elasto-lung point in both static and dynamic scans.

## 2. Materials and Methods

This was a prospective, multicenter, observational study conducted from March 2022 to December 2022. The study involved the following centers: Interventional Pulmonology Unit, IRCCS Azienda Ospedaliero-Universitaria di Bologna, Policlinico Sant’Orsola-Malpighi; Respiratory Unit, Azienda Ospedaliero-Universitaria di Modena, Policlinico di Modena; Pulmonology and Thoracic Endoscopy Unit, Azienda Ospedaliero-Universitaria di Parma; Interventional Pulmonology Unit, Azienda Ospedaliero-Universitaria San Luigi Gonzaga–Orbassano (TO); Pulmonology Unit, Azienda Ospedaliero Universitaria Maggiore della Carità di Novara; Pulmonology Unit, Ospedale dell’Angelo di Mestre–ULSS 3 Serenissima; and Interventional Pulmonology Unit, Fondazione Policlinico Universitario Agostino Gemelli IRCCS, Università Cattolica del Sacro Cuore. The target number of patients to be enrolled in each center was ten. Due to the logistical needs of the study, only the Interventional Pulmonology Unit of the IRCCS Azienda Ospedaliero-Universitaria of Bologna was able to enroll patients. The study was approved by the local ethical committee (number 655/2021/Oss/AOUBo), and patients provided written informed consent before enrollment. 

### 2.1. Patients

Consecutive patients undergoing CT scan-guided transthoracic needle aspiration for the diagnosis of lung lesions and aged more than 18 years were included in the study. Exclusion criteria were: severe coagulopathy, evidence of SARS-CoV-2 infection, presence of subpleural bullae (paraseptal emphysema), previous pleurodesis, and presence of pleural effusion. Severe coagulopathy was an exclusion criterion for undergoing a transthoracic CT biopsy, as it can induce an increased risk of bleeding complications during and after the transthoracic CT biopsy. Evidence of SARS-CoV-2 infection was also an exclusion criterion, as the biopsy procedure requires optimal respiratory conditions, which could be compromised in patients with an active infection. The presence of subpleural bullae (paraseptal emphysema), previous pleurodesis, and pleural effusion were further exclusion criteria, as these conditions may interfere with TUS and USE signals, making the detection of pneumothorax unreliable.

To minimize selection bias, a prospective design was used, recruiting consecutive patients undergoing CT scan-guided biopsies. This approach ensured that all eligible individuals were included, preventing arbitrary exclusions. The prospective design also reduced recall bias and ensured consistent and systematic data collection.

### 2.2. Lung Ultrasounds

**Figure 2:**
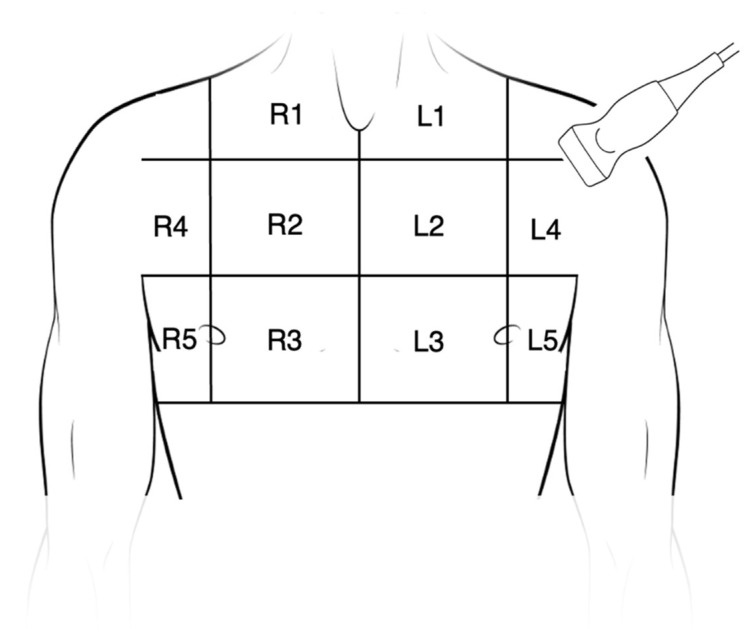
Anterior chest areas for LUS reporting (modified from reference [13]).

Immediately after undergoing a CT scan-guided transthoracic needle aspiration and the subsequent CT scan control in the supine position, each enrolled patient underwent a chest ultrasound. TUS was performed with the patient supine, using B-mode with a linear probe at 7.5 Megahertz (capturing both images and video clips), with subsequent activation of the elastographic mode (capturing additional images and video clips). The anterior thoracic regions were explored (Figure 2), according to the topographic division described by Zanforlin A. et al. [13]. A Hitachi HI VISION Preirus ultrasound system was used. Both B-mode and elastographic-mode images/videos were preset to: dynamic tissue harmonic imaging (dTHI) and HI COM active, gain 20, focus on the pleural line, and depth almost 2 cm under the pleural line.

To acquire the ultrasound and the elastography scans, the linear probe was positioned transversely to include only the intercostal space within the study volume, excluding the visualization of the ribs. The parietal pleural line was centered in the middle of the scan, with the thoracic wall and the lung framed respectively in the upper and in the lower sides of the image. A dual-view screen was employed, displaying the B-mode ultrasound on one side and the elastography view on the other (Figure 1). Due to the use of first-generation elastography, gentle compressions were applied perpendicularly to the chest wall with the probe to generate the characteristic elastography color patterns on the ultrasound monitor.

**Figure 3:**
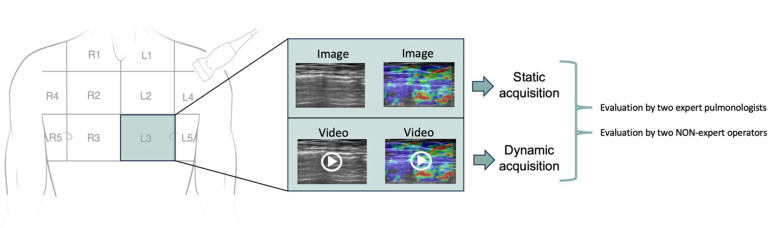
Ultrasound acquisition and evaluation. For each chest region, four acquisitions were made: one TUS image (static), one USE image (static), one TUS video (dynamic), and one USE video (dynamic). The resulting images (for static evaluation) and videos (for dynamic evaluation) were classified by anatomical region and then sent to two pulmonologists with expertise in chest ultrasound, as well as two non-expert operators (pulmonology/thoracic surgery fellows) for evaluation.

Ultrasound scans were performed by a physician experienced in both thoracic ultrasound and elastography. For each region of the chest, four acquisitions were recorded: one image from the TUS scan (static), one image from the USE scan (static), one video from the TUS scan (dynamic), and one video from the USE scan (dynamic) (Figure 3). The resulting images (for static evaluation) and videos (for dynamic evaluation) were categorized according to the anatomical regions and sent to two pulmonologists with expertise in chest ultrasound and two non-expert operators (pulmonology/thoracic surgery fellows). These physicians independently and blindly evaluated each iconographic study (and video clips of the B-mode scans only and those with the superimposed elastographic study), judging whether the specific displayed image or video was indicative of pneumothorax, normal lung, lung point, elasto-lung point, or doubtful.

To resolve any doubtful interpretation results, the sum of all evaluations, rather than relying solely on the agreement between individual evaluators, was considered. Specifically, aggregated data were derived from the evaluations of four different operators: two pulmonologists with expertise in chest ultrasound and two non-expert operators. This approach resulted in a total of 128 evaluations (4 evaluators assessing 32 patients) for each side.

The radiologist also assessed the presence or absence of pneumothorax in each topographic area evaluated by ultrasound and elastography, based on the control CT scans performed immediately after the biopsy. During the evaluation of the CT images, the radiologist did not have access to the ultrasound images.

### 2.3. Data Analysis

Mean values, standard deviations, and absolute and relative frequencies were used as descriptive statistics. To evaluate the diagnostic accuracy, sensitivity, specificity, and their 95% confidence intervals (95% CI) were estimated. The differences in the values of sensitivity and specificity were tested with a test on the equality of two proportions. To assess the between-rater agreement, the Cohen’s kappa coefficient, together with 95% CI, was used. The statistical analyses were performed using STATA 18, considering two-tailed *p*-values < 0.05 statistically significant. CT scans were used as the gold standard in the identification of pneumothorax.

Artificial intelligence (ChatGPT) was utilized for language editing of the abstract of this manuscript.

## 3. Results

Thirty-two patients were included in the study from March to December 2022. Data of the study population are described in Table 1. The majority were peripheral lung nodules adjacent to the pleura (40.6%), 25% were peripheral lung nodules distant to the pleura, 25% were peripheral masses adjacent to the pleura, and 9.4% were peripheral masses not adjacent to the pleura, central masses, or ground glass lesions not adjacent to the pleura. Final diagnosis was adenocarcinoma in 43.8% of cases, squamous cell carcinoma in 6.2%, metastasis of other cancers in 18.8%, benign features in 18.8%, and lymphoma in 3.1%. About 9% of cases showed no specific findings. Thirteen patients developed pneumothorax (40.63%), of which 18.75% were on the left side and 21.88% were on the right side.

Table 2 shows, for each scan mode (B-mode image, elastographic-mode image, B-mode video, elastographic-mode video), the presence or not of pneumothorax as compared to the gold standard. 

The presence of pneumothorax in one area (R1–R5 on the right or L1–L5 on the left) was sufficient to diagnose pneumothorax on the same side. The absence of vertical artifacts or sliding, the presence of lung point/elasto-lung point, and USE crossing the pleural line were all considered signs of pneumothorax. The data shown in the table refer to aggregated data derived from the evaluations of four different operators (both the two pulmonologists with expertise in chest ultrasound and the two non-expert operators), resulting in 128 total evaluations (4 evaluators for 32 patients) for each side.

Table 3 shows sensitivity, specificity, and positive and negative predictive values of the static evaluations (images) of the B-mode and elastographic mode and their comparison.

The same analysis is reported in Table 4 regarding the dynamic evaluations (videos).

The elastographic-mode image had significantly higher sensitivity and higher positive and negative predictive values, compared to the B-mode image, in identifying pneumothorax, using CT scans as the gold standard (sensitivity 76.9% vs. 21.2%, *p*-value < 0.001; positive predictive value 67.8% vs. 52.4%, *p*-value 0.01; negative predictive value 82.6% vs. 61.7%, *p*-value < 0.001).

The sensitivity of the elastographic-mode videos did not significatively differ from the sensitivity of B-mode videos (sensitivity 76.9% vs. 71.2%, *p*-value 0.5).

Table 5 shows an analysis of the sensitivity and specificity of videos in individual areas according to the judgment of the expert evaluators. R5 was not assessable, as four patients with pneumothorax at the CT scan were not found by the two experienced evaluators, neither with B-mode nor with USE. Concentrating on the L5 area, the sensitivity for B-mode was comparatively low at 16.7%, and the sensitivity for USE was also modest at 50%, with high variability.

Cohen’s kappa coefficients between expert and non-expert evaluators for each scan mode are reported in Table 6. The concordance of elastographic-mode images between expert and non-expert was significantly higher than that of B-mode images (*p* < 0.001). On the right side, the concordance of B-mode images could not be evaluated, since no pneumothorax was detected by any expert evaluator.

## 4. Discussion

This study investigated prospectively for the first time the different performances of TUS versus USE in detecting pneumothorax, using CT scans as the gold standard, in consecutive patients undergoing CT scan-guided transthoracic needle aspiration.

This study shows that the elastographic-mode image had a significatively higher sensitivity and higher positive and negative predictive values compared to the B-mode image in identifying pneumothorax (sensitivity 76.9% vs. 21.2%, *p*-value < 0.001; positive predictive value 67.8% vs. 52.4%, *p*-value 0.01; negative predictive value 82.6% vs. 61.7%, *p*-value < 0.001). This suggests that lung elastography better recognizes and reports pneumothorax on static images as compared to ultrasound.

To date, among all signs of pneumothorax, the most specific is lung point (specificity approaching 100% and sensitivity of 75%) [6], which is a dynamic sign. No studies have demonstrated the presence of a corresponding static sign with equal of better sensitivity and specificity. Even with the introduction of M-mode for pneumothorax evaluation, the use of static scans has not been widely implemented. Moreover, some authors suggest that M-mode may be unnecessary, as less experienced operators might face challenges in accurately positioning the ultrasound probe [14].

Additionally, the study demonstrated a higher concordance between expert and non-expert evaluations of elastographic-mode images, as compared to B-mode ones (even if this result is limited to one side), suggesting an easier interpretation of elastographic images. Taken together, these data suggest a potential role of the lung elastography image in detecting pneumothorax. Due to this, elastographic images could be potentially attached in a medical report, even if more data are needed to standardize this technique.

Concerning videos, the aggregate data showed that the sensitivity of the elastographic-mode videos was generally higher compared to B-mode videos, although the difference was not statistically significant (Table 4). The lack of statistical significance in the difference in sensitivity between USE and dynamic TUS may be explained by the fact that the lung point, which is a pathognomonic sign of pneumothorax on ultrasound, is inherently dynamic. Therefore, a possible explanation is that in dynamic evaluation, USE does not significantly enhance the sensitivity of dynamic TUS as it might with static TUS. This is likely why the difference in sensitivity between USE and dynamic TUS did not reach statistical significance.

The analysis for each individual areas showed non-unique behavior (Table 5). This could be due to the movement of the lung during the TUS and USE, compared to the CT scan, by definition a static exam. In particular, it can be noted that for R5, sensitivity could not be calculated because the four patients with pneumothorax identified on the CT scan were not detected by the two experienced evaluators, either with B-mode or with USE. Focusing on the L5 area, the sensitivity for B-mode was relatively low, compared to the other areas (16.7%), and the sensitivity for USE was also not high (50%), with wide variability. According to our data, both L5 and R5, on the left and right sides, did not appear to be easily assessable areas for the ultrasound diagnosis of pneumothorax (whether TUS or USE). It is unclear whether this low sensitivity was due to random factors or whether the position of these regions, lateral to the base of the hemithorax, potentially affected by diaphragmatic movements, made them more difficult to assess and interpret. Further studies with a larger sample size could help clarify this issue.

The use of elastography in the diagnosis of pneumothorax was first described in 2020 in a study investigating the feasibility of USE as a tool for confirming pneumothorax after detecting the lung point during TUS [12]. In that study, the elasto-lung point was detected in every patient with lung point. In our study, the gold standard for comparison in diagnosing pneumothorax was the chest CT scan, which allowed for the calculation of sensitivity, specificity, and both positive and negative predictive values for the two methods. Our study also included non-expert subjects, revealing a high concordance between expert and non-expert subjects, suggesting improved interpretability.

In this study, ultrasound and elastography images and videos were blindly evaluated by both experienced and non-experienced operators. This approach undoubtedly enhanced the objectivity of the evaluation, reducing the influence of clinical and radiological data. However, it should also be considered that, since ultrasound and elastography are dynamic bedside examinations, the data analysis could be partially influenced by the absence of clinical and radiological information. 

Therefore, TUS vs. USE should be investigated in a “real-life” context, where the operator both acquires the scan, evaluates the images/videos, and makes the diagnosis.

Although the study was designed to be multicentric, logistical issues led to it being monocentric, which may have affected the reproducibility of the data. Despite this limitation, the involvement of four evaluators helped to mitigate this bias.

Another limitation of the study was the absence of a pre-calculated sample size, which was not determined due to the feasibility nature of the study. However, to compensate for this, 32 patients were enrolled at a single center, surpassing the initial target of 10 patients per center. In addition, a post hoc power analysis was conducted: a sample size of 32 achieved 77% power to detect a change in sensitivity in one group from 0.70 (under the null hypothesis) to 0.200 (under the alternative hypothesis). The sensitivity in the other group was assumed to be 0.70 in both cases. The statistical test employed was a two-sided Z test with pooled variance, with a targeted significance level of 0.0500 and an actual achieved significance level of 0.046. Additionally, the prevalence of the disease (or condition of interest) was settled at 0.45.

In summary, our study suggests a potential application of elastography images in medical reports, recording the presence of a pneumothorax. This potential is supported by elastography’s feasibility for clinical use, given its non-invasive, safe nature and its ability to provide real-time imaging without the need for radiation or contrast agents. Moreover, its portability, cost-effectiveness, and ease of integration into existing ultrasound machines make it an accessible and practical tool for a wide range of healthcare settings. Although our data showed good concordance between expert and non-expert evaluators, further studies are needed to determine the number of procedures required to achieve significant concordance among non-expert operators.

Since this is the first prospective study on the use of elastography in patients with pneumothorax, several additional study scenarios could be explored. More studies are needed to investigate the potential role of dynamic USE in the evaluation of pneumothorax. Future studies could explore the use of ultrasound (USE) to diagnose pneumothorax in settings beyond post-biopsy, such as in emergency situations. With the growing availability of portable ultrasound, the application of this USE (if accessible) could also be examined in out-of-hospital settings to assess acute dyspnea in patients when radiology services are not available. Additionally, studies could investigate the effectiveness of USE in diverse patient populations and its diagnostic accuracy, compared to other imaging methods, in these settings.

## 5. Conclusions

In this observational monocentric study, it was demonstrated that thoracic USE can be useful as a photo to report the presence of pneumothorax. Although it has been shown that videos with USE tend to increase the sensitivity in the diagnosis of pneumothorax, this trend was not found to be statistically significant. Further multicenter studies using other types of USE could be useful to better define the potential of this technology in pneumothorax diagnosis, particularly in emergency settings. At present, based on the results of this study, USE could be useful for the diagnosis of pneumothorax only if already available.

## Figures and Tables

**Table 1:** Descriptive analysis.

	N (%)
**Age (years): mean (SD)**	68.56 (11.27)
**Gender**	
Male	12 (37.5%)
Female	20 (62.5%)
**Smoking habit**	
Never	9 (28.1%)
Former	11 (34.4%)
Active	12 (37.5%)
**Emphysema**	2 (6.2%)
**History of lung cancer**	1 (3.1%)
**History of neoplasia**	14 (43.8%)
**Biopsy side**	
Left	17 (53.1%)
Right	15 (46.9%)
**Post-biopsy symptoms**	
No symptoms	31 (96.9%)
Hemoptysis	1 (3.1%)

**Table 2:** Aggregated analysis of findings detected by both expert and non-expert evaluators.

	CT Scan Left		CT Scan Right	
	No Pneumothorax	Pneumothorax	No Pneumothorax	Pneumothorax
**B-mode image**				
No pneumothorax	95 (91.3%)	18 (75.0%)	94 (94.0%)	24 (85.7%)
Pneumothorax	9 (8.7%)	6 (25.0%)	6 (6.0%)	4 (14.3%)
**Elastographic-mode image**				
No pneumothorax	86 (82.7%)	6 (25.0%)	87 (87.0%)	7 (25.0%)
Pneumothorax	18 (17.3%)	18 (75.0%)	13 (13.0%)	21 (75.0%)
**B-mode video**				
No pneumothorax	87 (83.7%)	7 (29.2%)	93 (93.0%)	9 (32.1%)
Pneumothorax	17 (16.3%)	17 (70.8%)	7 (7.0%)	19 (67.9%)
**Elastographic-mode video**				
No pneumothorax	80 (76.9%)	6 (25.0%)	81 (81.0%)	7 (25.0%)
Pneumothorax	24 (23.1%)	18 (75.0%)	19 (19.0%)	21 (75.0%)

**Table 3:** Aggregated analysis of sensitivity, specificity, and positive and negative predictive values of static TUS and USE evaluations. Statistically significant results are denoted by an asterisk (*).

	B-Mode Image	Elastographic-Mode Image	*p*-Value
Sensitivity	21.2	76.9	<0.001 *
Specificity	86.8	75.0	0.06
Positive Predictive value	52.4	67.8	0.01 *
Negative Predictive value	61.7	82.6	<0.001 *

**Table 4:** Aggregated analysis of sensitivity, specificity, and positive and negative predictive values of dynamic TUS and USE evaluations.

	B-Mode Video	Elastographic-Mode Video	*p*-Value
Sensitivity	71.2	76.9	0.50
Specificity	77.6	64.5	0.07
Positive predictive value	68.5	59.7	0.14
Negative predictive value	79.7	80.3	0.90

**Table 5:** Sensitivity and specificity of videos in the analysis for each individual area among expert evaluators compared to CT scan.

	Sensitivity		Specificity	
Topographic Division	B-Mode Video	Elastographic-Mode Video	B-Mode Video	Elastographic-Mode Video
**L1**	75%	75%	94.6%	92.9%
**L2**	70%	80%	98.1%	96.3%
**L3**	62.5%	87.5%	62.5%	100%
**L4**	75%	75%	95%	95%
**L5**	16.7%	50%	100%	100%
**R1**	100%	100%	91.9%	91.9%
**R2**	100%	100%	98.3%	96.6%
**R3**	50%	33.3%	100%	100%
**R4**	25%	25%	100%	96.4%
**R5**	-	-	-	-

**Table 6:** Cohen’s kappa coefficients between expert and non-expert evaluators in each scan mode. Statistically significant results are denoted by an asterisk (*).

	Cohen’s Kappa Coefficient		*p*-Value
	**B-mode image**	**Elastographic-mode image**	
Left	0.193	0.770	<0.001 *
Right	-	0.529	-
	**B-mode video**	**Elastographic-mode video**	
Left	0.396	0.415	0.9
Right	0.124	0.449	0.644

## Data Availability

The data presented in this study are available upon request from the corresponding author due to privacy reasons.
